# EXPLORING AGE, GENDER, AND RELATIONSHIP CLOSENESS DIFFERENCES IN RESPONSES TO IGNORED DISRESPECT

**DOI:** 10.1093/geroni/igz038.1118

**Published:** 2019-11-08

**Authors:** Jenessa C Steele, Amanda Chappell, Rachel Scott

**Affiliations:** Radford University, Radford, Virginia, United States

## Abstract

Emotional responses to disrespect tend to be negative (Hawkins, 2015). Little is known about how responses to disrespect vary across age groups and relationship closeness. It is unknown whether older adults have more emotional protection against disrespectful experiences, or are more deeply affected due to relationship closeness. Overall, we might expect that older adults react less negatively to disrespect compared to young adults, as they are more-skilled emotion regulators (Carstensen, 1991; English & Carstensen, 2014). We aimed to explore if, and under which circumstances, older adults are more or less sensitive to disrespect compared to younger adults. Three hundred participants responded to six scenarios illustrating ignored disrespect. Participants were randomly assigned to close or distant relationship disrespect scenarios. Relationship closeness was first determined by requesting participants identify a person in each layer of Kahn and Antonucci’s (1980) Social Convoy Model. Identified names were then automatically inserted into the six scenarios. Emotional responses and sensitivity to each scenario were recorded. Participants in the close condition reported more sensitivity to disrespect and negative emotions than participants in the distant condition. Females reported more sensitivity to disrespect and negative emotions than males. We did not find overwhelming support for age differences in responses to disrespect. A single scenario indicated younger participants more sensitive to disrespect than older participants. Findings suggest it is more hurtful to be disrespected by someone close to you and females may be more sensitive to disrespect than males. More research investigating the role of age in disrespect is needed.

